# Semantic-Aware Fusion Network Based on Super-Resolution

**DOI:** 10.3390/s24113665

**Published:** 2024-06-05

**Authors:** Lingfeng Xu, Qiang Zou

**Affiliations:** 1 School of Microelectronics, Tianjin University, Tianjin 300072, China; xu_1998@tju.edu.cn; 2 Tianjin International Joint Research Center for Internet of Things, Tianjin 300072, China; 3 Tianjin Key Laboratory of Imaging and Sensing Microelectronic Technology, Tianjin University, Tianjin 300072, China

**Keywords:** infrared and visible image fusion, super-resolution network, multi-modal features, semantic-aware fusion network

## Abstract

The aim of infrared and visible image fusion is to generate a fused image that not only contains salient targets and rich texture details, but also facilitates high-level vision tasks. However, due to the hardware limitations of digital cameras and other devices, there are more low-resolution images in the existing datasets, and low-resolution images are often accompanied by the problem of losing details and structural information. At the same time, existing fusion algorithms focus too much on the visual quality of the fused images, while ignoring the requirements of high-level vision tasks. To address the above challenges, in this paper, we skillfully unite the super-resolution network, fusion network and segmentation network, and propose a super-resolution-based semantic-aware fusion network. First, we design a super-resolution network based on a multi-branch hybrid attention module (MHAM), which aims to enhance the quality and details of the source image, enabling the fusion network to integrate the features of the source image more accurately. Then, a comprehensive information extraction module (STDC) is designed in the fusion network to enhance the network’s ability to extract finer-grained complementary information from the source image. Finally, the fusion network and segmentation network are jointly trained to utilize semantic loss to guide the semantic information back to the fusion network, which effectively improves the performance of the fused images on high-level vision tasks. Extensive experiments show that our method is more effective than other state-of-the-art image fusion methods. In particular, our fused images not only have excellent visual perception effects, but also help to improve the performance of high-level vision tasks.

## 1. Introduction

In the field of multi-modal image fusion, infrared and visible image fusion has been the mainstream direction. An infrared image is an image acquired by an infrared sensor by detecting light in the infrared radiation range, which can fully show the thermal distribution of an object but is less visually appealing and contains fewer texture details. While visible images are images acquired by visible sensors by detecting light in the visible range, this kind of image usually contains rich texture and structural information, but in low light and complex environments such as rain and fog, the image quality is poor, and the target information is not clear enough. Therefore, infrared and visible images are fused to make full use of their spatio–temporal correlation and complementary information. The fused images are more informative than unimodal signals, not only containing salient targets and rich texture details, but they can also be more effectively used in high-level vision tasks such as object recognition [1], target detection [2], tracking [3], and semantic segmentation [4].

There are more current infrared and visible image fusion methods, but they are mainly categorized into two groups: traditional methods and deep learning (DL)-based methods. Traditional fusion methods are usually based on fusion in the spatial and transform domains [5], and the image fusion frameworks used mainly include multi-scale transform (MST)-based fusion frameworks [6,7,8,9,10,11,12,13,14,15], sparse representation (SR)-based fusion frameworks [16,17,18], subspace-based fusion frameworks [19,20,21], saliency-based fusion frameworks [22], and hybrid fusion frameworks [23,24,25]. And according to the adopted network architecture, the DL-based image fusion methods can be mainly categorized into three groups, which are autoencoders (AE)-based image fusion frameworks [26,27,28,29,30], convolutional neural network (CNN)-based image fusion frameworks [31,32,33,34,35,36,37] and generative adversarial network-(GAN) based image fusion frameworks [38,39,40,41,42,43].

In recent years, although DL-based image fusion methods have made significant progress and the fused images satisfy various requirements, there are still many challenges in the field of image fusion. On the one hand, most of the current studies are limited to single-task fusion models and overemphasize the visual quality of the fused images, without sufficiently considering whether the fused images can subsequently effectively improve the performance of high-level vision tasks. Therefore, we adopt a semantic-aware fusion network [44], which not only improves the quality of the fused images, but also meets the performance requirements of subsequent high-level vision tasks. Specifically, we introduce a segmentation network to predict the segmentation results of the fused images and utilize the network to construct the semantic loss. The semantic loss is then utilized to guide the training of the fusion network via backpropagation, forcing the fused images to contain more semantic information. Finally, we adopt a task-driven evaluation approach to evaluate the performance of the fused images by evaluating their performance on the semantic segmentation task.

On the other hand, there are more low-resolution images in the available datasets due to hardware limitations of equipment such as digital cameras and interference from various complexities of weather and light levels. Low-resolution images are often accompanied by loss of detail and structural information, as well as loss of spatial coherence. The loss of detail and structural information reduces the quality of fused images, while the loss of spatial coherence may lead to spatial discontinuity or unnaturalness in the fusion result, which in turn affects the visual effect and realism of the fused images. To address these issues, we propose a semantic-aware fusion framework based on super-resolution. Specifically, we utilize a super-resolution network to enhance the quality and details of the source images, which are then fed into a semantic-aware fusion network for fusion to obtain higher quality fused images. In addition, due to the obvious differences between visible and infrared light images in terms of color, texture, contrast, etc., it may be that the same super-resolution network has a better super-resolution effect on infrared images and a poor super-resolution effect on visible images. To address this problem, we design a multi-branch hybrid attention module (MHAM) in the super-resolution network to enhance the network’s ability to extract multi-modal features. The MHAM strengthens the description of multi-modal features through a multi-channel attention module and a multi-space attention module, thus effectively improving the quality of super-resolution images.

Meanwhile, it has been shown [45] that infrared and visible images share the same scene, and they have statistical commonality in low-frequency information, such as background and large-scale environmental features; however, their information in the high-frequency portion is relatively independent, such as the texture information in the visible modality and the temperature information in the infrared modality, both of which are unique to their respective modalities. In order to efficiently and comprehensively extract cross-modal features, we design a comprehensive information extraction module (STDC) in the fusion network. The STDC module consists of a Swin Transformer hybrid depth-wise convolution (Dwconv). Dwconv is used to mine low-frequency localized features in the source image, and it can dramatically reduce the number of parameters and the computational effort as compared to the ordinary convolution [46]. Swin Transformer, on the other hand, is not only able to effectively extract high-frequency global features in the source image, but also saves computational resources when processing high-resolution images compared to the traditional Transformer [47]. This combination not only improves the efficiency of processing high-resolution images, but also more comprehensively fuses the fine-grained complementary information in infrared and visible images, thus generating fused images with excellent visual perception. 

In summary, the primary contributions of this study are outlined as follows:

- A novel semantic-aware fusion framework for infrared and visible images based on super-resolution has been ingeniously crafted by integrating super-resolution networks, fusion networks, and segmentation networks. This framework not only excels in image fusion but also achieves outstanding performance in high-level vision tasks.
- In the fusion network, a comprehensive information extraction module is designed, aiming at efficiently processing high-resolution images and achieving more comprehensive fine-grained complementary feature extraction.
- In the super-resolution network, a multi-branch hybrid attention module is designed to endow the network with strong cross-domain adaptation capability. This means that excellent super-resolution reconstruction results can be achieved even when dealing with cross-modal image super-resolution.

The rest of the paper will be organized as follows. In Section 2, we will briefly introduce related work on image super-resolution, image fusion, and task-driven algorithms. In Section 3, we will present the details of the proposed semantic segmentation fusion framework based on super-resolution, including the analysis of the problem, the design of the loss function, the construction of the network architecture, and the formulation of the training strategy. In Section 4, we compare the performance of the proposed method with other alternatives and demonstrate the significant advantages of the method in this paper. Finally, in Section 5, we will summarize the whole study.

## 2. Related Works

In this section, we provide a comprehensive overview of the background information and related work that are highly pertinent to the methodology proposed in this paper. This includes discussions on algorithms for infrared and visible image fusion, image super-resolution techniques, and task-driven low-level vision algorithms.

### 2.1. Image Fusion Algorithms

#### 2.1.1. Traditional Image Fusion Methods

The key to traditional image fusion algorithms lies in feature extraction and fusion, which mainly uses relevant mathematical transformations to manually analyze the activity level in the spatial or transform domain and design fusion rules. Typical traditional image fusion frameworks can be mainly categorized into multi-scale transform (MST)-based fusion frameworks, sparse representation (SR)-based fusion frameworks, subspace-based fusion frameworks, saliency-based fusion frameworks, and hybrid fusion frameworks. Among them, the multi-scale transform-based fusion framework is mainly a method to decompose the source image into different scales of information and analyze and fuse them at these scales. Commonly used multi-scale transform methods include wavelet transforms [6,7,8] (e.g., discrete wavelets), pyramid transforms [9,10,11,12] (e.g., gaussian pyramid or laplace pyramid), and methods based on multi-scale geometric analysis [13,14,15] (e.g., contour transform or curve transform). These transform methods are able to capture both detailed and global information in an image and have different applicability to different types of images. The sparse representation-based fusion framework, on the other hand, builds a joint sparse representation matrix of features after extracting the features from the source image, and this sparse representation matrix is the result of the fusion of multiple features [18]. The advantage of this framework lies in the flexible extraction and fusion of image features through appropriate selection of basis functions and optimization algorithms.

In the subspace-based fusion framework, the source image is viewed as a set of points in a high-dimensional space, and this space can be decomposed into multiple subspaces, each representing a specific structure or feature in the image [19]. The framework requires efficient subspace decomposition algorithms and suitable fusion strategies to ensure the quality and efficiency of the fusion results. In addition, the saliency-based fusion framework focuses on extracting salient structures, regions, and objects in the source image through visual saliency maps (VSMs) [22]. This framework is effective in avoiding loss of contrast and obtaining a better overall appearance of the fused image.

In addition, some other scholars have successfully combined the advantages of different frameworks through different perspectives and proposed hybrid models to achieve better image fusion performance. Li et al. [23] proposed a hybrid multi-resolution approach by combining the wavelet transform and contour transform to solve the problem of limited performance enhancement of the single wavelet- or contour-wave based methods. And then, Wang et al. [24] proposed an image fusion method based on non-subsampled contour wavelet transform (NSCT) and sparse representation to address the inherent shortcomings of multi-scale transform and sparse representation methods. In addition, Liu et al. [25] proposed a generalized image fusion framework based on Multi-scale Transform (MST) and Sparse Representation (SR), where the source image is first decomposed using MST, and then the low-frequency components are fused using SR, which successfully combines the respective advantages of MST and SR and achieves improved performance of the fused image.

Although traditional fusion methods may perform well in specific tasks and scenarios, they usually require manual adjustment of parameters or selection of appropriate methods to adapt to different problems. In addition, traditional fusion methods cannot fully take into account the characteristics of different modal images, and the fusion rules are usually simple rules designed manually and the fusion strategy is so coarse that the fusion performance is very limited. Therefore, deep learning is expected to fuse infrared and visible images.

#### 2.1.2. AE-Based Image Fusion Methods

The fusion method based on autoencoders (AE) involves training autoencoders on a large-scale image dataset, followed by merging them according to specific fusion rules such as element-wise summation, and finally, the fused image is reconstructed from the merged features using an autoencoder [26]. Li et al. [27] were pioneers in applying deep learning to the fusion of infrared and visible images, proposing a straightforward AE-based image fusion framework that cascades encoders, a fusion layer, and decoders in sequence. Initially, encoders extract image features, then additive and L1 norm strategies are designed in the fusion layer, with the final image reconstruction performed by decoders. Despite its relatively rudimentary network structure and the simplistic manually designed fusion strategies at the fusion layer, this framework sets a foundational reference for subsequent researchers in the field of visible and infrared fusion (VIF).

To address the limitations of manually designed fusion strategies, Xu et al. [28] introduced an AE-based image fusion method that leverages classifier saliency (CS). This method first classifies infrared and visible source images using a classifier, then assesses the importance of each pixel based on its impact on the classification results to create a saliency map, which is then used to merge feature maps. Furthermore, to thoroughly extract the complementary information from infrared and visible images, Xu et al. [29] also developed a decomposition method that applies disentangled representation to the fusion of infrared and visible images, allowing the images to be processed separately through respective encoders to produce representations related to scene and sensor modalities. Additionally, aiming to generate natural and information-rich fused images, Liu et al. [30] introduced an end-to-end visible and infrared image fusion framework based on autoencoders, consisting of encoders, a residual fusion module (RFM), and decoders. The RFM, crafted from spatial pyramid designs based on dilated convolution, replaces manually designed fusion rules in the fusion layer to extract and merge multi-scale features from source images, resulting in a fusion outcome that maintains more details and thermal radiation information from the original images.

#### 2.1.3. CNN-Based Image Fusion Methods

CNN-based image fusion frameworks achieve end-to-end image fusion by designing network structures and loss functions, thus avoiding the tediousness of manually designing fusion rules [31]. In order to cope with different types of fusion tasks, Zhang et al. [32] proposed a typical CNN-based generalized image fusion framework (IFCNN), which selects specific fusion rules, such as elementwise-max, elementwise-min, and elementwise-mean, for different types of images to improve the generalization ability of the fusion framework. Considering the complexity of manual fusion rule design, Long et al. [33] proposed an unsupervised visible (VIS)/infrared (IR) fusion network (RXDNFuse) based on an aggregated residual dense network. RXDNFuse is designed as an end-to-end model that utilizes two loss function strategies to optimize the similarity constraints and network parameter training. The key to this method is to transform the image fusion problem into a structure-to-intensity ratio preservation problem for IR/VIS images. And then, in order to fully preserve the thermal targets in IR images and the texture structure in visible images, Ma et al. [31] proposed an infrared and visible image fusion network (STDFusionNet) based on salient target detection, which utilizes a salient target mask to annotate the regions that are of more concern to human beings or machines in the IR images, and combines this salient target mask with the design of a loss function to guide the feature extraction and reconstruction, in order to realize the feature extraction network to selectively extract salient target features from infrared images and background texture features from visible images.

In addition, for the existing image fusion algorithms that do not consider the light factor in the modeling process, Tang et al. [34] proposed a progressive image fusion network (PIAFusion) based on light perception, which uses a light perception sub-network to estimate the light distribution and compute the light probability, and then constructs a light perception loss through the light probability to guide the fusion network’s training in order to achieve the source image’s public information and complementary information which are fully fused. Immediately after, Tang et al. [35] also proposed a darkness-free infrared and visible image fusion method (DIVFusion) to design the scene illumination de-entanglement network (SIDNet) and texture-contrast enhancement fusion network (TCEFNet), respectively, in order to remove illuminance degradation and fuse the complementary information in the nighttime visible images. Meanwhile, in response to the existing CNN fusion methods that directly ignore remote dependencies, Rao et al. [36] proposed a transformer module-based fusion algorithm (TGFuse) for infrared and visible images, which utilizes the transformer technique to learn effective global fusion relations, interacting shallow features extracted by CNNs, as well as refining spatially scoped and cross-channel fusion relations. Furthermore, in order to achieve full alignment of infrared and visible images, Li et al. [37] proposed a new alignment-free fusion method for infrared and visible panning images, which transforms the image alignment problem into a feature alignment problem in an end-to-end framework. A cross-modulation strategy is utilized for dynamic alignment of features so as to adaptively measure the spatial correlation of displacements and dynamically extract the aligned features.

#### 2.1.4. GAN-Based Image Fusion Methods

The Generative Adversarial Network (GAN)-based image fusion approach is to model the image fusion problem as an adversarial game problem between a generator and a discriminator. Ma et al. [38] pioneered the introduction of GAN into the field of image fusion by creatively adopting a GAN-based image fusion framework (FusionGAN). The method establishes an adversarial game between a generator and a discriminator, and then utilizes the generator to produce a fused image with primary infrared intensity and additional visible gradients, and finally forces the fused image to have more details present in the visible image through the discriminator. And later, Ma et al. [39] also proposed an end-to-end infrared and visible image fusion model based on detail-preserving adversarial learning, based on FusionGAN, to design the detail loss and target edge enhancement loss to improve the quality of detail information and edge sharpening of the fused image. In addition, in order to cope with the limitation of the single discriminator function, Ma et al. [40] proposed a dual discriminative conditional generative adversarial network (DDcGAN), which establishes an adversarial game between a generator and two discriminators to achieve the distinction of structural differences between the fused image and the two source images.

Meanwhile, in order to solve the problem of the loss of luminance and detail information in the fusion of infrared and visible images, Zhang et al. [41] designed a full-size jump connection and double Markov discriminator (GAN-FM). This method utilizes a full-size jump connection generator to extract and fuse depth features at different scales, and then estimates the probability distributions of infrared and visible modalities by a bi-Markov discriminator to achieve greater retention of valid information in infrared and visible images. Subsequently, Wang et al. [42] proposed a robust cross-modal generative alignment paradigm, which utilizes a cross-modal perceptual style transfer network (CPSTN) to transform cross-modal image alignment into unimodal alignment, in order to solve the problem of the fused image’s degraded quality when the multi-modal data are not aligned. In addition, in order to equip the fused image with high color fidelity, Yue et al. [43] proposed a new method based on the diffusion model (Dif-Fusion), which is used to generate the distribution of the multi-channel input data in order to improve the ability of multi-source information aggregation and the color fidelity.

### 2.2. Image Super-Resolution

Image super-resolution (SR) methods aim to recover a given low-resolution image into a corresponding high-resolution image. In order to address the limitations of the single-image super-resolution based on traditional methods, Dong et al. [48] proposed for the first time a deep learning method for single-image super-resolution based on CNNs, which is utilized to directly learn the end-to-end mapping between low/high-resolution images. The performance of this method was dramatically improved compared to the traditional methods, but due to the use of a shallow network, the sensory field of the network was small and could not extract the global feature information in the image. And then, Zhang et al. [49] introduced the channel attention mechanism into image super-resolution for the first time, which utilizes the interdependence between channels and adaptively readjusts the channel intelligent features to enhance the characterization ability of CNN. 

In addition, Deng et al. [50] proposed a deep coupled feedback network (CF-Net) to address the problem of greater low dynamic range (LDR) and low resolution (LR) of images in existing datasets. The key to this method is to utilize the intrinsic complementarity and correlation that exists between the multi-exposure image fusion (MEF) and super-resolution (SR) methods, and then the multi-exposure image fusion network and the super-resolution network are jointly trained in order to achieve the purpose of the two promoting each other, so as to achieve MEF and SR at the same time. In addition, in order to solve the problem of the structural redundancy of the image super-resolution network, Chen et al. [51] proposed a lightweight single-image super-resolution network that incorporates multi-level features. The method employs two layers of nested residual blocks to better extract features and reduce the number of parameters, thus improving the speed of network training convergence.

In addition, in the hyperspectral image (HSI) reconstruction task, for the phenomenon that the best model validated on the simulated dataset has a failure in real measurements, Li et al. [52] proposed a novel reference-free HSI quality assessment metric through ranked feature learning (R-NHSIQA). The metric realizes the acquisition of the best model in real-world tasks by calculating the Wasserstein distance between the depth feature distribution of the reconstructed HSI and the reference distribution. To address the lack of a fast, objective and comprehensive image quality assessment (IQA) method for unmanned aerial hyperspectral images (UAV-HSIs), Tian et al. [53] proposed a multi-feature based fuzzy comprehensive evaluation (FCE) method. The method selected four radiometric features, three spatial features and two spectral features to construct the indicator set, and finally realized a comprehensive evaluation of UAV hyperspectral image quality by establishing a fuzzy evaluation threshold table and an affiliation function.

In addition, an ongoing problem in remote sensing image processing is how to assess the quality of fused images obtained through different methods. To address this problem, Wang et al. [54] constructed a full-resolution remote sensing image quality assessment method based on the combination of low-level features and deep-level features. The method extracts low-level features and deep-level features from fused images, and after statistical analysis and feature combination, the final quality score is predicted using the random forest regression method. In addition, in the field of face super-resolution (FSR), FSR may alter the identity of an individual or introduce artifacts that affect recognizability, yet existing image quality assessment (IQA) methods are not yet able to assess this problem well. Chen et al. [55] used a benchmark dataset and a simplified reference quality metric to subjective and objective assessment of FSR-IQA to effectively address such problems. 

Furthermore, in order to efficiently solve the image super-resolution task, Zhou et al. [56] proposed a dynamic recursive process by exploring the parameter sharing mechanism on the transformer blocks. The method utilizes the recursive image super-resolution transformer (RIST) to share weights among different blocks, thus achieving effective model size reduction and gradual image restoration.

Multispectral (MS) images have high spatial resolution, in contrast to hyperspectral (HS) images, which have lower spatial resolution but more accurate spectral resolution. However, due to the low spatial resolution, the utilization of HS data in order to obtain an acceptable signal-to-noise ratio for the final product is highly limited. Therefore, fusion of these two types of images is expected to improve the resolution of HS. In particular, in order to have both high spatial resolution and images with rich spectral information, various fusion methods and algorithmic improvements have been explored in detail to improve the quality and accuracy of the fused images by Vivone et al. [57]. In addition, Vivone et al. describe open problems and provide guidance for future research.

Low-resolution images are often accompanied by loss of detail and structural information, which seriously affects the accuracy and visual effect of image fusion. A single fusion network has limited enhancement effect in processing these low-resolution source images, limiting the subsequent applications of fused images. Given the prevalence of low-resolution images in existing datasets, we introduce a super-resolution network to enhance the quality and details of the source images so that the fusion network can integrate the source image features more accurately, thus obtaining higher quality fused images.

### 2.3. Task-Driven Low-Level Vision Algorithms

Given that most of the existing research is limited to making the fusion network generate images that match human visual perception, ignoring the fact that the significance of image fusion lies in facilitating the performance of high-level vision tasks (e.g., semantic segmentation, etc.), some studies have started to explore task-driven fusion methods. In particular, Tang et al. [44] took a new perspective on the image fusion task and proposed a semantic-aware real-time image fusion network (SeAFusion), which utilizes high-level vision tasks to drive image fusion. Specifically, a segmentation network is introduced to predict the segmentation results of the fused images, and the fusion and segmentation networks are jointly trained. The optimization of the fusion network is jointly constrained by the content loss generated by the fusion network and the semantic loss generated by the segmentation network to generate fused images that are both consistent with human visual perception and conducive to high-level vision tasks.

There are also studies on image fusion driven by target detection. Among them, Liu et al. [58] proposed a target-aware fusion dual adversarial learning (TarDAL) network, which employs a single generator and a dual discriminator in order to fully preserve the complementary information of the images, while the fusion network is motivated by a target detection task to generate fused images with high detection accuracy. In addition, Sun et al. [59] proposed a detection-driven fusion network for infrared and visible images (DetFusion). The method employs the joint training of a target detection network and an image fusion network to utilize the detection loss to guide multi-modal image fusion in order to produce fused images that contribute to target detection performance.

Based on the above existing techniques, this paper designs a super-resolution based semantically aware infrared and visible image fusion model. The fused images generated by this model not only help in high-level vision tasks (e.g., semantic segmentation), but also have excellent visual perception.

## 3. Proposed Methods

In this section, we comprehensively describe the framework for super-resolution-based semantically aware infrared and visible image fusion, including the problem formulation of the framework, content loss and semantic loss. At the same time, we delve into the structure of the multi-branch hybrid attention module in super-resolution networks and the architecture of the fusion network based on the comprehensive information extraction module.

### 3.1. Problem Formulation

In source images with poor resolution, the shapes and contours of categories such as bicycles, pedestrians, and guardrails appear blurred or distorted, significantly diminishing the visual quality and realism of the fused images. To address this issue, we have designed a super-resolution network to enhance the quality and detail of the source images.

**Figure 1:**
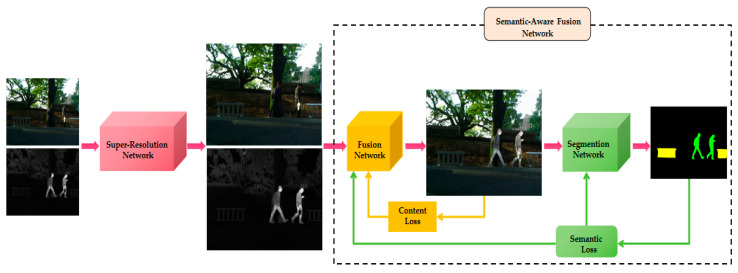
The overall framework for super-resolution-based semantic-aware infrared and visible image fusion algorithm.

Given a pair of aligned visible images $I_{ir}\in R^{H\times W\times 3}$ and infrared images $I_{\mathrm{vi}}\in R^{H\times W\times 1}$, the quality of the fused images $I_{f}\in R^{H\times W\times 3}$ generated in the fusion network often depends on the customized loss function. To achieve high-quality fusion, we use content loss and semantic loss to jointly constrain the optimization of the fusion network. The overall framework of the super-resolution-based semantic-aware infrared and visible image fusion algorithm is shown in Figure 1.

First, we design a multi-branch hybrid attention module (MHAM) in a super-resolution network to efficiently handle the modal differences between visible and infrared images. We apply MHAM to extract the rich fine-grained detail features of infrared and visible images in channel and space, which can be represented as follows:

(1) $$F^{\prime }=M_{C}\left(F\right)⨂F⨂M_{C}(F)$$

(2) $$F^{\prime \prime }=M_{S}(F^{\prime })⨂F^{\prime }⨂M_{S}(F^{\prime })$$

where *F* denotes the features of a visible image or an infrared image, $F\in R^{H\times W\times C}.$ ⨂ denotes multiplication by bit. $M_{C}(F)$ denotes the channel attention feature map and $M_{S}(F^{\prime })$ denotes the spatial attention feature map. MHAM sequentially infers a 1D channel attention map $M_{C}\in R^{1\times 1\times C}$ and a 2D spatial attention map $M_{S}\in R^{H\times W\times 1}$. Equation (1) represents the optimization of the input feature F by multiplexed channel attention module to obtain the new feature $F^{\prime }$. Equation (2) represents the optimization of the input $F^{\prime }$ by multiplexed spatial attention module to obtain the final fine-grained detailed feature $F^{\prime \prime }$. The channel attention module focuses the correlation between different channels in the feature map, which can be expressed as follows:

(3) $$M_{C}(F)=\sigma (\mathrm{MLP}(\mathrm{AvgPool}(F))+\mathrm{MLP}(\mathrm{MaxPool}(F)))$$

where σ denotes the sigmoid function, and MLP(AvgPool(F)) and MLP(MaxPool(F)) denote the average pooling and maximum pooling operations performed on the input feature map F, respectively, and processed by the multi-layer perceptual machine (MLP) to obtain the attentional weights for each channel. Then the MLP output features are elementally summed before sigmoid activation to obtain the final single-branch channel attention feature $M_{C}(F)$. And then, the two-way channel attention features are multiplied with the input features for adaptive feature optimization to obtain the optimized features $F^{\prime }$. Compared to the single-branch channel attention module, the advantage of the dual-branch channel attention module is that it can identify and focus on important channel features more effectively, thus significantly improving the representation of the feature map.

The spatial attention module focuses on the correlation between different spatial locations in the feature map, which can be expressed as follows:

(4) $$M_{S}(F^{\prime })=\sigma (f^{7\times 7}([\mathrm{AvgPool}(F^{\prime });\mathrm{MaxPool}(F^{\prime })])$$

where $f^{7\times 7}$(∙) denotes a convolution operation with a filter size of 7 × 7. The two-way spatial attention feature is multi-plied with the input feature $F^{\prime }$ to obtain the final fine-grained detail feature $F^{\prime \prime }$. In this way, the network is able to focus on the detailed features at different spatial locations in the image, thus improving the quality of the image.

**Figure 2:**
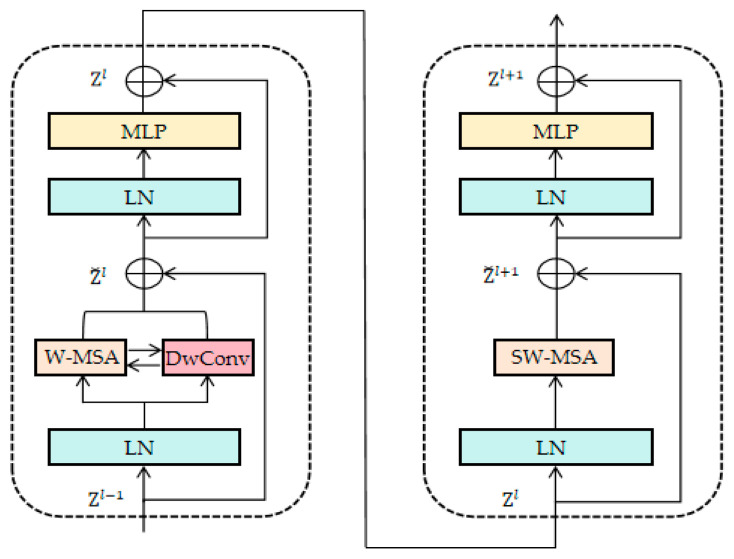
Internal structure of the STDC module.

**Figure 3:**
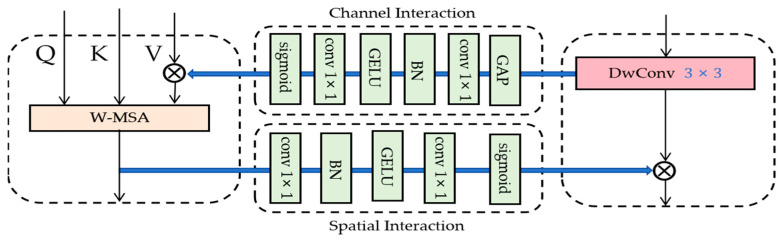
Detailed design of W-MSA bi-directional interaction with Dwconv.

In addition, in order to fully extract the complementary information of infrared and visible images, the STDC module is designed in the fusion network. More specifically, a form of window-based multi-head self-attention (W-MSA) interacting with depth-wise convolution (Dwconv) in both directions is designed inside the Swin Transformer in order to realize the full extraction of global and local detail information, while also efficiently processing high-resolution images. The process of extracting complementary information by the STDC module is represented as follows:

(5) $${\overset{˘}{Z}}^{l}=\mathrm{MIX}(W\text{-}\mathrm{MSA}(\mathrm{LN}(Z^{l-1})),\mathrm{Dwconv}(\mathrm{LN}(Z^{l-1})))+Z^{l-1}$$

(6) $$Z^{l}=\mathrm{MLP}(\mathrm{LN}({\overset{˘}{Z}}^{l}))+{\overset{˘}{Z}}^{l}$$

(7) $${\overset{˘}{Z}}^{l+1}=S\text{-}\mathrm{WMSA}(\mathrm{LN}(Z^{l}))+Z^{l}$$

(8) $$Z^{l+1}=\mathrm{MLP}(\mathrm{LN}({\overset{˘}{Z}}^{l+1}))+{\overset{˘}{Z}}^{l+1}$$

where $Z^{l-1}$ denotes the features of infrared and visible images, and LN denotes LayerNorm layer which is used to normalize the input features. W-MSA is used to capture the information of the local region. Dwconv is used to extract the features efficiently. Equation (6) indicates that after normalization of ${\overset{˘}{Z}}^{l}$, the feature transformation is performed by MLP and added to the input ${\overset{˘}{Z}}^{l}$ to obtain the updated feature $Z^{l}$. SW-MSA denotes shift-window based multi-head self-attention for capturing a wider range of features. Pairing W-MSA and SW-MSA together can effectively save computation when processing high-resolution images [47]. $Z^{l+1}$ denotes the multi-modal information output from the STDC module. MIX denotes the feature mixing function that realizes the bidirectional interactions between the W-MSA block and the Dwconv block. The MIX function firstly projects the input features onto the parallel branches through the normalization layer. Then it mixes the complementary features of the source image following the steps shown in Figure 2 and Figure 3.

In W-MSA, the image is partitioned into multiple small windows and the self-attention computation is performed independently within each window. Since the size of each window is much smaller than the entire image, this greatly reduces the number of elements involved in each computation, and thus the overall computation is significantly reduced. Combining W-MSA and Dwconv allows for adequate and efficient extraction of local features from the source image. In SW-MSA, the window is misaligned horizontally or vertically (e.g., shifted half a window’s distance to the right and downward) so that the window coverage area overlaps with a portion of the neighboring window, thus realizing cross-window information. Ultimately, this design not only improves processing efficiency, but also enhances the model’s ability to capture global and local information.

Meanwhile, considering the requirements of high-level vision tasks on fused images, semantic loss is employed to guide the fusion network to retain the semantic information in the source image to a greater extent. More specifically, the segmentation network $N_{s}$ is added to segment the fusion result $I_{f}\in R^{H\times W\times 3}$. The gap between the segmentation result $I_{s}\in R^{H\times W\times 3}$ and the semantic label $L_{s}\in {\left(1,C\right)}^{H\times W}$ is denoted as the semantic loss $L_{\mathrm{semantic}}$, where H and W are the height and width of the source image, respectively, and *C* denotes the number of object categories in the source image. The semantic segmentation process can be represented as follows:

(9) $$I_{s}=N_{s}(I_{f})$$

The size of the semantic loss $L_{\mathrm{semantic}}$ can reflect the richness of the semantic information contained in the fused image, which can be expressed as follows:

(10) $$L_{\mathrm{semantic}}=E(I_{s},L_{s})$$

where E(·) denotes the error function.

### 3.2. Loss Function

Our framework aims to generate fused images that are consistent with human visual perception and can effectively improve the performance of high-level vision tasks. We refer to Tang et al. [44] to introduce content loss $L_{\mathrm{content}}$ and semantic loss $L_{\mathrm{semantic}}$ to jointly constrain the optimization of the fusion network. The role of content loss is to guide the fusion network to fully integrate the complementary information of the source images, such as texture details in the visible image and salient targets in the infrared image, so as to ensure that the fused images remain visually natural and realistic. The semantic loss, on the other hand, guides the training of the fusion network by means of backpropagation to ensure that the generated fused images contain more semantic information required for high-level vision tasks, which helps to improve the accuracy and efficiency of subsequent tasks.

#### 3.2.1. Content Loss

The content loss $L_{\mathrm{content}}$ is used to respond to the size of the difference between the fused image $I_{f}$ and the source image and consists of intensity loss $L_{\mathrm{int}}$ and texture loss $L_{\mathrm{texture}}$. The specific representation is as follows:

(11) $$L_{\mathrm{content}}=L_{\mathrm{int}}+\alpha L_{\mathrm{texture}}$$

where α is used to regulate the weighting coefficients of the relative importance of intensity loss $L_{\mathrm{int}}$ and texture loss $L_{\mathrm{texture}}$.

Intensity loss $L_{\mathrm{int}}$ mainly employs pixel-level differences to measure the luminance or intensity difference between the fused image and the source image for the purpose of constraining the overall apparent intensity of the fused image. $L_{\mathrm{int}}$ can be defined as follows:

(12) $$L_{\mathrm{int}}=\frac{1}{\mathrm{HW}}{\left\|I_{f}-\max(I_{\mathrm{ir}},I_{\mathrm{vi}})\right\|}_{1}$$

where H and W are the height and width of the source image, respectively, and ${\left\|\cdot \right\|}_{1}$ denotes the $l_{1}$ paradigm. max(·) denotes the element-level maximum selection for integrating the pixel intensity distribution of the source image.

And the texture loss $L_{\mathrm{texture}}$ is used to direct the fused image to include finer-grained texture details, which can be defined as follows:

(13) $$L_{\mathrm{texture}}=\frac{1}{\mathrm{HW}}{\left\|\left|\nabla I_{f}\right|-\max(\left|\nabla I_{\mathrm{ir}}\right|,\left|\nabla I_{\mathrm{vi}}\right|)\right\|}_{1}$$

where ∇ denotes the Sobel gradient operator, which is used to measure the fine-grained texture information of an image. $\left|\cdot \right|$ denotes the absolute value operation. Here, the optimal texture of the fused image is the largest set of infrared and visible image textures; for a detailed description refer to the original paper [44].

The results show that the fusion network based on the STDC module, guided by content loss, not only maximizes the acquisition of intensity distribution, but also fully preserves the fine-grained detail information of the source image, thus generating fused images with superior visual perception.

#### 3.2.2. Semantic Loss

Semantic loss assumes a two-fold task. On the one hand, it guides the segmentation network to output superior segmentation results. On the other hand, it joins hands with the content loss to jointly guide the optimization of the fusion network to ensure that the generated fused images not only remain visually natural and realistic, but also have rich semantic information. The semantic loss consists of the main semantic loss $L_{\mathrm{main}}$ and the auxiliary semantic loss $L_{\mathrm{aux}}$. The definitions of main semantic loss and auxiliary semantic loss can be expressed as follows:

(14) $$L_{\mathrm{main}}=\frac{-1}{\mathrm{HW}}\sum_{h=1}^{H}\sum_{w=1}^{W}\sum_{c=1}^{C}L_{\mathrm{so}}^{\left(h,w,c\right)}\log(I_{s}^{\left(h,w,c\right)})$$

(15) $$L_{\mathrm{aux}}=\frac{-1}{\mathrm{HW}}\sum_{h=1}^{H}\sum_{w=1}^{W}\sum_{c=1}^{C}L_{\mathrm{so}}^{\left(h,w,c\right)}\log(I_{\mathrm{sa}}^{\left(h,w,c\right)})$$

where $I_{s}\in R^{H\times W\times 3}$ denotes the segmentation result and $I_{\mathrm{sa}}\in R^{H\times W\times 3}$ denotes the auxiliary segmentation result. $I_{\mathrm{so}}\in R^{H\times W\times 3}$ denotes the unique heat vector transformed from the segmentation label $L_{s}\in {\left(1,C\right)}^{H\times W}$. Primary semantic loss and auxiliary semantic loss guide the fused images from different perspectives to retain the semantic information required for high-level vision tasks. Ultimately, the definition of semantic loss can be expressed as follows:

(16) $$L_{\mathrm{semantic}}=L_{\mathrm{main}}+\lambda L_{\mathrm{aux}}$$

where λ is used as a constant to balance the primary and auxiliary semantic losses, which is taken as 0.1 in reference to the original article [60].

Finally, the joint loss used to guide the training of the fusion network is defined as follows:

(17) $$L_{\mathrm{joint}}=L_{\mathrm{content}}+\beta L_{\mathrm{semantic}}$$

where β is a hyperparameter characterizing the importance of the semantic loss $L_{\mathrm{semantic}}$. To balance the low-level and high-level tasks, we iteratively train the fusion network and the segmentation network. Then, all parameters in the fusion network are optimized using the Adam optimizer under the guidance of the joint loss. Meanwhile, β is gradually increased with iterative training so that the segmentation network better fits the fusion model. the definition of β is denoted as follows:

(18) β=γ×(y−1)

where γ is a constant used to balance the semantic loss and content loss, and y denotes the *y*th iteration. As the number of iterations increases, the semantic loss increases gradually, thus guiding the training of the fusion network more accurately.

### 3.3. Super-Resolution Network Framework

**Figure 4:**
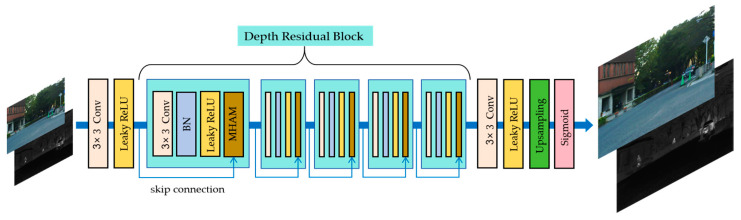
MHAM-based super-resolution network architecture for infrared and visible images.

In order to solve the problem of existing datasets with more images with blurred picture quality, we introduce a super-resolution network to enhance the quality and details of the source images, which are then fed into the fusion network for effective fusion. Meanwhile, considering the differences of multi-modal images, a multi-branch hybrid attention module (MHAM) is designed in the super-resolution network to enhance the network’s ability to characterize multi-modal features. The MHAM-based super-resolution network for infrared and visible images is shown in Figure 4.

As shown in Figure 4, the super-resolution network consists of two parts: feature extraction and image reconstruction. The feature extraction part includes a convolutional layer with a kernel size of 3 × 3, a Leaky ReLU activation layer, and a depth residual block. Among them, the convolutional layer and Leaky ReLU activation function are used to extract shallow features of the source image. By using multiple deep residual blocks, it aims to extract finer-grained features from the shallow features while ensuring the stability of the whole network.

The image reconstruction part consists of a convolutional layer with a kernel size of 3 × 3, a Leaky ReLU activation layer, an upsampling layer, and a sigmoid activation layer. After the feature map output from the residual module passes through the convolutional layer and the Leaky ReLU activation layer, it is enlarged by two times the size in the upsampling layer, and then passes through the sigmoid activation layer to obtain the final high-quality and high-resolution image required.

**Figure 5:**
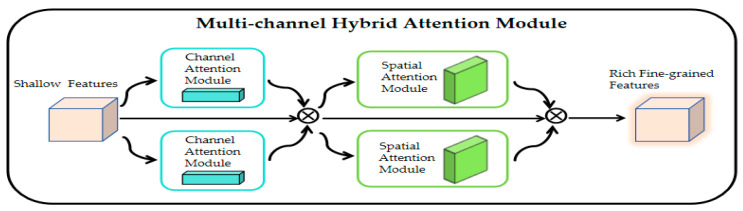
Detailed design of the MHAM module.

The inclusion of a batch normalization layer in the residual module is to address the problem of information loss. In particular, the Multi-branch Hybrid Attention Module (MHAM) is designed to realize the network’s effective processing of multi-modal images. The design of the MHAM module is shown in Figure 5. The MHAM module draws on the idea of CBAM [61] (Convolutional Block Attention Module) by adding a one-way channel attention module and a one-way spatial Attention Module, and such a design aims to obtain richer fine-grained features, thus increasing the network’s ability to understand the variability of multi-modal features.

**Figure 6:**
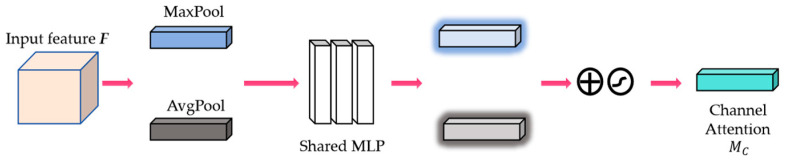
Internal structure of the channel attention module.

**Figure 7:**
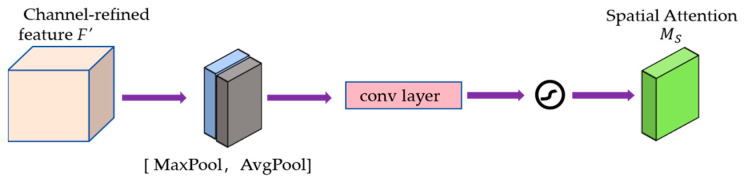
The inner structure of the spatial attention module.

As can be observed from Figure 5, the shallow features of the source image are multiplied bitwise with the output features passing through the two channel attention modules in the multiplier to obtain the channel attention features; then the channel attention features are multiplied bitwise with the output features passing through the two spatial attention modules in the multiplier to finally obtain the rich fine-grained features. Meanwhile, the internal detailed constructions of the channel attention module (CAM) and spatial attention module (SAM) are shown in Figure 6 and Figure 7, respectively. Specifically, the shallow features *F* of the source image are fed into the shared multi-layer perceptron (MLP) for processing after average pooling and maximum pooling operations to obtain the attention weights of each channel. Then the features output from the MLP are subjected to elemental summation followed by sigmoid activation to obtain the channel attention feature map $M_{C}$. Next, the channel attention feature map is input to the spatial attention module, which firstly, also undergoes the average pooling and maximum pooling operations, and then undergoes the convolution operation with a kernel size of 7 × 7, and finally, the sigmoid activation function to obtain the final spatial attention feature map $M_{S}$.

### 3.4. Fusion Network Framework

**Figure 8:**
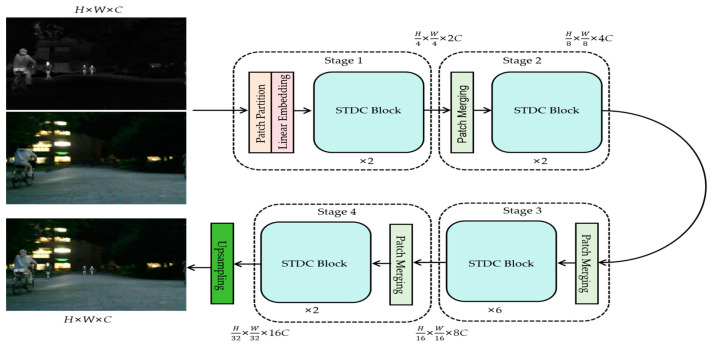
STDC-based network architecture for infrared and visible image fusion.

In order to comprehensively acquire the fine-grained complementary information of the source image, as well as to efficiently process high-resolution images, we propose an STDC-based fusion network for infrared and visible images, as shown in Figure 8. The fusion network consists of a feature extractor and an image reconstructor, where the feature extractor contains a patch segmentation layer, a linear embedding layer, a comprehensive information extraction module, and a patch merging layer.

As shown in Figure 8, the linear embedding layer is used to do a linear transformation of the channel data of each pixel, which specifies that each 4 × 4 neighboring pixel is a Patch, and the source image of H×W×C size will obtain a feature map of H4×W4×2C size after passing through the patch partition layer and the linear embedding layer. Observation shows that except for Stage 1 in which the patch partition layer and linear embedding layer are passed first, the remaining three Stages are first downsampled through the patch merging layer. Among them, the height and width of the feature map will be halved, and the depth will be doubled after each downsampling through the patch merging layer. The designed STDC module can not only accelerate the training speed of the network, but also fully obtain the complementary information in the infrared and visible images. The specific design of the STDC module is shown in Figure 2. The STDC module consists of the Swin Transformer block and the Dwconv block (depth-wise convolution). Among them, the Swin Transformer block includes a normalization layer, a multi-layer perceptual machine layer (MLP), a W-MSA block (window-based multi-head self-attention), and a SW-MSA block (shifted window-based multi-head self-attention).

After downsampling by the patch merging layer, the feature map is divided into multiple disjoint regions (window), and then each window is processed by using W-MSA and SW-MSA in pairs, which also effectively reduces the amount of computation in the face of high-resolution images. A detailed description of W-MSA and SW-MSA can be found in the original paper [47]. And the introduction of the Dwconv block in the Swin Transformer block aims to flexibly utilize the respective advantages of Swin Transformer and depth-wise convolution in order to achieve the effective extraction of global and local information of the source image. Meanwhile, the W-MSA block and the Dwconv block are designed to interact with each other in a bidirectional form, and the detailed design is shown in Figure 3.

In Figure 3, the Dwconv block and the W-MSA block interact bi-directionally through channel/spatial interaction, the local information extracted by the Dwconv block is input to the multiplier for feature fusion and enhancement with the help of channel interaction with the feature map, and the output is sent to the W-MSA block for windowing, and the global features output from the W-MSA block are input to the multiplier for feature fusion and enhancement with the help of spatial interaction with the local features. The global features from the W-MSA block are then fed into the multiplier with the help of spatial interaction to fuse and enhance the local features. This design aims to fully utilize the interactions between the various modules in order to achieve adequate extraction of fine-grained complementary information from the source image, so that the fusion network can generate fused images with superior visual perception.

## 4. Results

In this section, we provide experimental configurations and implementation details, and describe our approach in detail. Then, we validate the superiority of the approach through comparison and generalization experiments. Meanwhile, we further conduct task-driven evaluation experiments of different fusion methods from the perspective of high-level vision tasks to gain a deeper understanding of the impact of various fusion strategies on model performance. Finally, we conducted a number of ablation experiments to demonstrate the effectiveness of our specific designs, including the incorporation of super-resolution networks, the design of a multi-branch hybrid attention module, and the design of a comprehensive information extraction module.

### 4.1. Experimental Configurations

We compare our approach with seven state-of-the-art methods, including two AE-based methods, namely RFN-Nest [62] and DenseFuse [27], two GAN-based methods, namely AttentionFGAN [63] and Dif-Fusion [43], two CNN-based methods, namely U2Fusion [64] and CDDFuse [45], and a high-level task-driven approach, namely SeAFusion [44]. The implementations of all these methods are publicly available and we conducted experiments following the parameter settings in the original paper. Also, in order to fully evaluate the proposed algorithms, we conducted extensive qualitative experiments on the MSRS [34], RoadScene [32] and TNO [65] datasets.

In addition, six statistical evaluation metrics, namely entropy (EN) [66], mutual information (MI) [67], visual information fidelity (VIF) [68], spatial frequency (SF) [69], standard deviation (SD), and $Q_{\mathrm{abf}}$, were selected to quantitatively assess the quality of the fused images. EN was used to measure the amount of information contained in the fused images, while MI was used to measure the degree of information overlap between the source image and the fused image. Both of them evaluate the fusion performance from an information theoretic point of view. VIF evaluates the information fidelity of the fused image from the point of view of the human visual system. SF is used to measure the spatial frequency information contained in the fused image. SD statistically reflects the distribution of the pixel values and the contrast ratio of the fused image. $Q_{\mathrm{abf}}$ is used to evaluate the effect of the edge information transfer from the source image to the fused image. Better fusion results are obtained when fusion algorithms with higher values of EN, MI, VIF, SF, SD and $Q_{\mathrm{abf}}$ are used.

### 4.2. Implementation Details

We train the semantic-aware fusion network on the MSRS dataset. The MSRS dataset consists of a total of 1444 pairs of infrared and visible images, where the training set contains 1083 pairs of images while the test set contains 361 pairs of images. The MSRS dataset provides semantic labels for nine objects, namely, car, people, bike, curve, car stop, guardrail, color cone, bump and background. Also, all images are normalized to [0, 1] before inputting into the network.

To balance the low and high-level tasks, we perform joint iterative training of the fusion and segmentation networks and set the number of iterations Y to 5, epoch to 100, and the total number of steps is set to 100,000. We set the hyperparameter α, which balances texture loss and intensity loss, to 2, 6, and 10 for our experiments, and found that *α* = 10 maximizes the preservation and enhancement of image texture details while maintaining overall image intensity consistency. In addition, the value of the hyperparameter *β*, which characterizes the importance of semantic loss, gradually increases as training proceeds, which implies that the semantic loss will have an increasing weight in the total loss. Setting the constant γ, which balances the semantic and content losses, to 1 ensures that this increase is linear and stable, avoiding excessive fluctuations. Therefore, we set the constant γ, which balances the semantic loss and content loss, to 1, and the hyperparameter α, which is used to balance the texture loss and the intensity loss, to 10, to ensure that the weight of each term is appropriately weighted in the loss function. 

When performing joint iterative training, we borrowed the idea of Tang et al. [44] to set the parameter size. In the subsequent experimental analysis, we validate the chosen parameter sizes to illustrate the superiority of the chosen parameters. We choose the Adam optimizer with a batch size of 8, $\beta_{1}$ of 0.9, $\beta_{2}$ of 0.99, weight decay of 0.0002, and an initial learning rate of 0.002 to optimize the fusion network. The initial learning rate is set to 0.002, which is more moderate to ensure that the network can reduce the loss function value faster in the initial stage, and at the same time to avoid the training instability caused by too large of a step size. $\beta_{1}$ and $\beta_{2}$ are momentum parameters in the Adam optimizer, which are used to compute the first-order moment estimation and the second-order moment estimation of the gradient. In general, $\beta_{1}$ is usually set to 0.9, which is mainly used to accelerate the convergence and avoid the local minimum problem. $\beta_{2}$ is usually set to a value close to 1 (e.g., 0.99), which is used to stabilize the training process and reduce the oscillation of the gradient by smoothing out the second-order moment estimates. A smaller batch size (8) was chosen for the joint training with the aim of providing more frequent updates.

Meanwhile, we optimize the segmentation network using small batch stochastic gradient descent with a batch size of 16, momentum of 0.9, and weight decay of 0.0005. Weight decay is a regularization technique that prevents overfitting by adding L2 paradigms of weights to the loss function. Smaller weight decay (e.g., 0.0002 and 0.0005) can improve the generalization ability of the model by preventing overweighting while maintaining model performance. Choosing a slightly larger batch size in stochastic gradient descent (16) aims to balance update frequency and stability. In addition, the initial learning rate is set to 0.01, and the learning rate update is the initial learning rate multiplied by ${(1-\frac{\mathrm{iter}}{\max_{\mathrm{iter}}})}^{\mathrm{power}}$, where power is set to 0.9 [60]. Our method is implemented on the PyTorch platform, and all experiments are performed on a 15-core AMD EPYC 7371 16-CoreProcessor CPU and an RTX A5000 GPU.

In addition, if the infrared image and the visible image are not aligned, the information of different bands will not be correctly superimposed, resulting in the loss of key details or misleading information, which cannot accurately reflect the real situation of the target object. This is specifically manifested in heavy shadows or blurring of the fused image, which affects the visual effect and the clarity of the image. Therefore, before the experiment, we performed alignment processing on the infrared and visible images to achieve more accurate feature extraction and fusion.

Furthermore, considering that both MSRS and RoadScene datasets contain color visible images, we adopt a borrowed strategy similar to the original paper [70] for processing. First, we convert the visible images to YCbCr color space. Then, the Y-channels of the visible and infrared images are fused using a fusion algorithm. Next, the fused image is converted to an RGB color space with the Cb and Cr channels of the visible image. Finally, the RGB fused image is input into the segmentation network for subsequent processing.

### 4.3. Comparative Experiment

We compared the proposed method with seven other algorithms on the MSRS dataset to fully evaluate the performance of our fusion network.

#### 4.3.1. Qualitative Results

**Figure 9:**
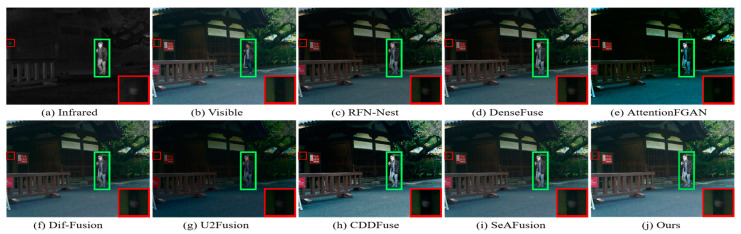
Qualitative comparison of our proposed method with seven state-of-the-art methods on MSRS dataset 00273D images.

**Figure 10:**
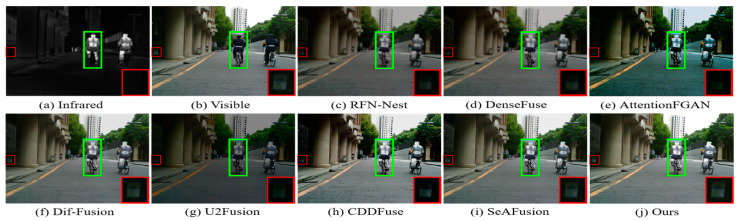
Qualitative comparison of our proposed method with seven state-of-the-art methods on MSRS dataset 00537D images.

**Figure 11:**
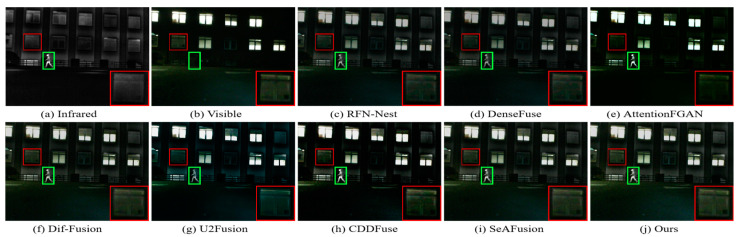
Qualitative comparison of our proposed method with seven state-of-the-art methods on MSRS dataset 00690N images.

**Figure 12:**
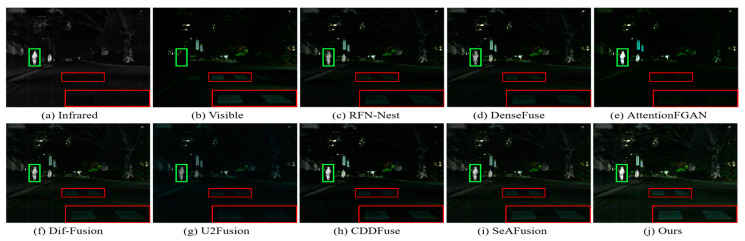
Qualitative comparison of our proposed method with seven state-of-the-art methods on MSRS dataset 00872N images.

The MSRS dataset covers two typical scenarios, daytime and nighttime, and in order to visualize the advantages of our fusion framework in generating fused images with superior visual perception, two daytime scenarios and two nighttime scenarios were selected for subjective evaluation. The visualization results are shown in Figure 9, Figure 10, Figure 11 and Figure 12. In the daytime scenes, the thermal radiation information of the infrared images can be utilized as supplementary information to the visible images. Therefore, a fused image with satisfactory visual quality should contain rich texture details in the visible image and enhance salient targets in the infrared image. As shown in Figure 9 and Figure 10, AttentionFGAN and U2Fusion perform poorly in preserving the texture details of the visible image, and the images synthesized by both of them are visually darker with greater color distortion. Although RFN-Nest and DenseFuse try to fuse the detail information of the visible image with the thermal radiation information of the infrared image, these two types of information are easily interfered by useless information during the fusion process, for example, the thermal radiation targets of both RFN-Nest and DenseFuse exhibit blurring. We zoom in on a region with a red box to illustrate the phenomenon of texture details being contaminated with varying degrees of spectral contamination. At the same time, the green box is used to highlight the problem of useless information weakening salient targets. Only our method, Dif-Fusion, CDDFuse, and SeAFusion, can preserve rich texture details while highlighting thermal radiation targets. Unfortunately, the texture details and contour details in the red zoomed-in region of Dif-Fusion, CDDFuse, and SeAFusion are blurred to varying degrees, and only our fused image displays the thermal radiation information and texture information clearly and distinctly. Therefore, only our method can effectively integrate the complementary information of the source images while ensuring the superior visual quality of the fused images.

In nighttime scenes, infrared and visible images usually provide only limited information about the scene. Therefore, it is a challenging task to adaptively integrate meaningful information from visible and infrared images. As shown in Figure 11 and Figure 12, we can see that all the methods fuse the complementary information in infrared and visible images to some extent, but there are still some subtle differences in the fusion results of different methods. In particular, there are different degrees of spectral contamination in the texture regions of AttentionFGAN and U2Fusion. Comparative observation of the texture information in the red zoomed-in region and the thermal radiation target in the green box shows that, except for our fusion, the other methods introduce more or less useless information into the fused images, which is manifested in the contamination or blurring of the texture information by noise, as well as the weakening of the salient targets. For example, RFN-Nest, DenseFuse, and U2Fusion exhibit dimming of thermal radiation targets, Dif-Fusion and SeAFusion show blurring of the red zoomed regions, and CDDFuse has the problem of darker hues in the red zoomed regions. 

It is worth emphasizing that the fused images generated by our fusion model contain clear and rich texture details, thanks to the prior quality and detail enhancement of the source images using a super-resolution network and the strong descriptive ability of the STDC module for fine-grained complementary information. Meanwhile, under the joint guidance of content loss and semantic loss, the fusion network is prompted to purposely fuse the meaningful information in the source image, thus generating a fused image with rich semantic information.

#### 4.3.2. Qualitative Results

**Figure 13:**
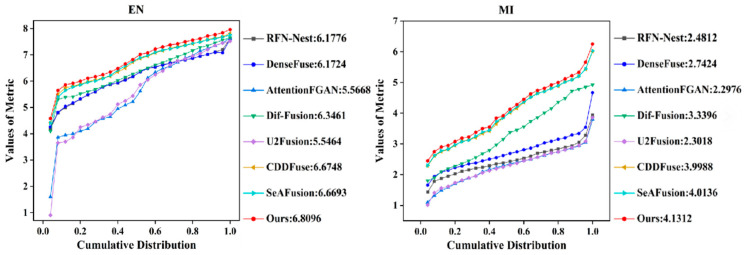

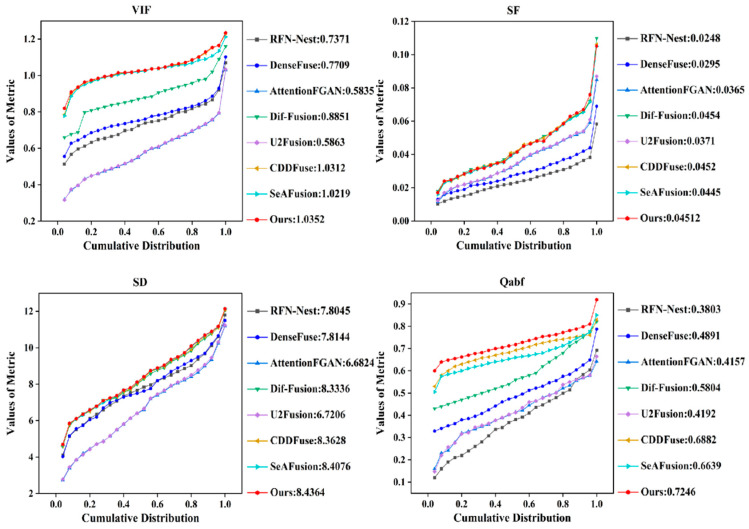
A quantitative comparison of 350 pairs of images from the MSRS dataset was performed for the six metrics EN, MI, VIF, SF, SD and $Q_{\mathrm{abf}}$. A point (*x*, *y*) on the curve denotes that there are 100 ∗ *x* percent of image pairs which have metric values no more than *y*.

We analyzed the quantitative results for 350 pairs of images using six statistical metrics as shown in Figure 13. It can be seen that our method shows significant advantages in five metrics, i.e., EN, MI, VIF, SD, and $Q_{\mathrm{abf}}$. The highest EN indicates that the fused image obtained by our method contains the most information. The best MI indicates that our method successfully transfers most of the information from the source image to the fused image. The best VIF result indicates that our fused image is more compatible with the human visual system. Meanwhile, our method obtains the best SD, which indicates that our fused image has the optimal contrast. In addition, our method has the best $Q_{\mathrm{abf}}$, which means that our fused image fully preserves the edge information in the source image, thanks to the super-resolution network’s enhancement of the clarity of the source image as well as the powerful fine-grained feature extraction capability of the STDC module. In terms of SF metrics, our proposed method is only slightly lower than CDDFuse.

### 4.4. Generalization Experiment

We conducted comparison tests with other methods using models trained on the MSRS dataset and directly on the RoadScene and TNO datasets to demonstrate the generalization performance of our proposed method.

#### 4.4.1. Qualitative Results

**Figure 14:**
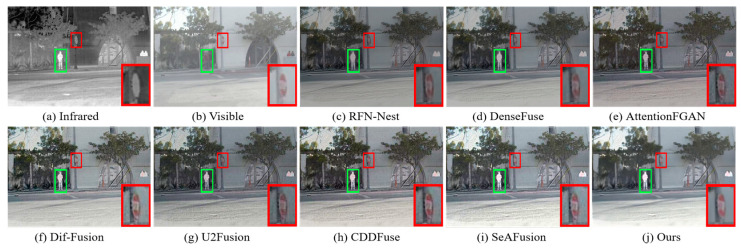
Qualitative comparison of our proposed method with seven state-of-the-art methods on RoadScene dataset FLIR_04598 images.

**Figure 15:**
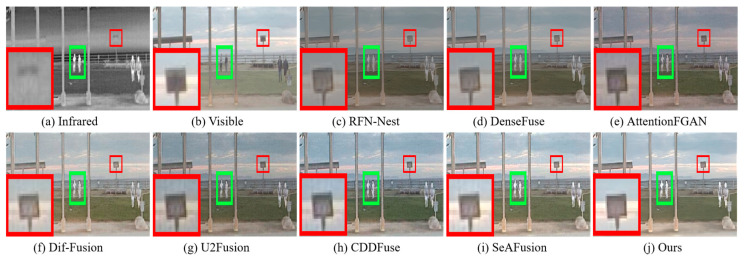
Qualitative comparison of our proposed method with seven state-of-the-art methods on RoadScene dataset FLIR_06621 images.

A qualitative comparison of the different algorithms on the RoadScene dataset is shown in Figure 14 and Figure 15. We zoom in on the texture details with the red box while highlighting the salient targets with the green box. We can observe that the texture region is contaminated with thermal radiation for almost all methods. In addition, RFN-Nest, DenseFuse, AttentionFGAN, and U2Fusion suffer from different degrees of spectral contamination, the fusion images are darker in color, and the intensity information of the salient targets of RFN-Nest, DenseFuse, and AttentionFGAN is weakened to different degrees. Although Dif-Fusion, CDDFuse and SeAFusion are disturbed by a small amount of useless information, the texture details show different degrees of blurring. From a visual perception point of view, our fused image is closest to the original visible image, and the pixel intensities of the salient targets are consistent with those of the infrared image. In particular, our texture detail regions show more clarity.

**Figure 16:**
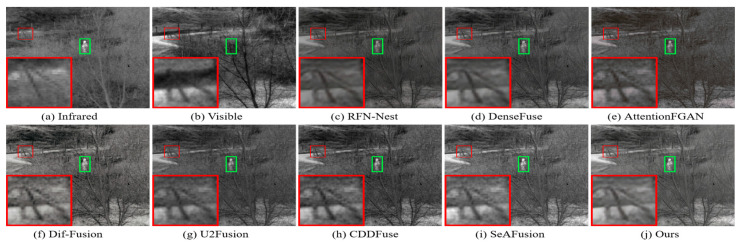
Qualitative comparison of our proposed method with seven state-of-the-art methods on TNO dataset sandpath_18 images.

**Figure 17:**
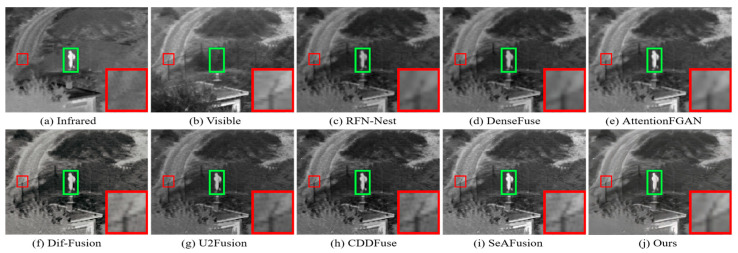
Qualitative comparison of our proposed method with seven state-of-the-art methods on TNO dataset Nato_camp_sequence_1819 images.

The visualization results of the different methods on the TNO dataset are shown in Figure 16 and Figure 17. As can be seen from the green and red boxes, the thermal radiation targets of RFN-Nest, DenseFuse and AttentionFGAN are severely weakened, and the edge details of RFN-Nest are extremely blurred. In addition, Dif-Fusion, CDDFuse, and SeAFusion suffer from more severe thermal radiation contamination and noise contamination, and the texture detail region has a large gap with the visible image. Only our method successfully preserves the texture details of the visible image while maintaining the intensity of the remarkable target.

#### 4.4.2. Qualitative Results

We also selected 25 pairs of images from each of the RoadScene and TNO datasets for quantitative evaluation to compare the performance of different methods on the six metrics, and the specific results are shown in Table 1 and Table 2. As can be seen in Table 1, our method ranks first in four metrics, namely, EN, VIF, SF, and $Q_{\mathrm{abf}}$, on the RoadScene dataset. This means that our fused image not only contains rich information and texture details, but also has the most spatial frequency information and excellent visual quality. Meanwhile, our method performs best in preserving the edge information in the source image. In addition, in the two metrics of MI and SD, our method only lags behind Dif-Fusion and CDDFuse by a slight margin, respectively, which indicates that our method is equally capable of transferring most of the information from the source image to the fused image.

As shown in Table 2, on the TNO dataset, our method outperforms the compared methods in terms of MI, VIF, SD, and $Q_{\mathrm{abf}}$. These results indicate that our fused images have richer information and texture details, superior contrast, and higher visual quality. There is only a small gap between our method and CDDFuse in the EN metric, and our method is only slightly behind Dif-Fusion in the SD metric.

It is noteworthy that our method ranks first in both VIF and $Q_{\mathrm{abf}}$ metrics on the MSRS, RoadScene, and TNO datasets. This signifies that our approach not only achieves exceptional visual perception in fused images but also effectively preserves the edge information of the source images. This advantage can be largely attributed to the enhancements in quality and detail provided by the super-resolution network. In the MSRS dataset, we selected visible images of a daytime and a nighttime scene; in the RoadScene dataset, a daytime visible image; and in the TNO dataset, a nighttime visible image, to demonstrate the super-resolution network’s ability to enhance the clarity of source images.

**Figure 18:**
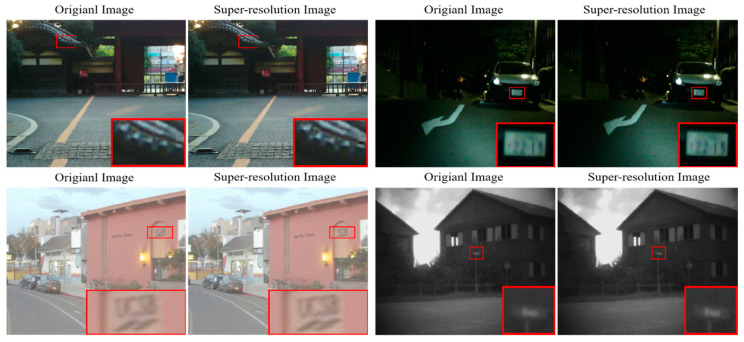
The upper left and upper right show the 00268D image and 01376N image from the MSRS dataset, respectively, while the lower left and lower right show the FLIR_06920 image and Marne_02_Vis image from the RoadScene and TNO datasets.

As shown in Figure 18, after the super-resolution network processing, we can clearly observe the following improvement effects: in the 00268D image, the outlines of the eaves become clearer and more accurate; the license plate numbers in the 01376N image and the letters of the English alphabet in the FLIR_06920 image become easier to distinguish; and the road sign information in the Marne_02_Vis image becomes clearer and more visible. Finally, these source images with enhanced quality and details are fed into the fusion network for processing, resulting in fused images with excellent visual perception and rich detail information.

#### 4.4.3. Efficiency Comparison

We selected 40 pairs of infrared and visible images from each of the MSRS, RoadScene and TNO datasets for fusion prediction and counted the mean and standard deviation of the running time (unit: second) of the different methods, and the results are shown in Table 3. It is observed that all the deep learning based fusion methods show efficient running efficiency, thanks to GPU acceleration. Our method has the fastest runtime on all three datasets, thanks to the advantages of Swin Transformer and Deep Separable Convolution in computational processing. Swin Transformer efficiently captures the global features of an image through a multi-head self-attention mechanism, while Deep Separable Convolution dramatically reduces the amount of computation and increases the processing speed. The combination of the two significantly improves the overall efficiency of the model.

In summary, our approach not only maintains high operational efficiency, but also generates high-quality fused images.

#### 4.4.4. Overfitting Analysis

**Figure 19:**
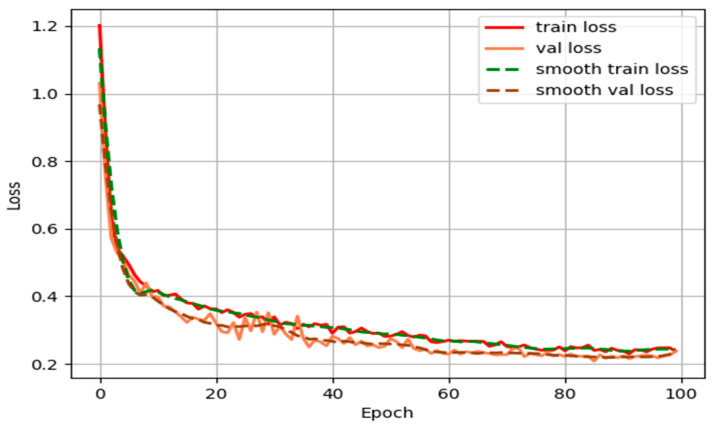
Loss function curve.

We show the variation of the curves of training loss and validation loss to try to explain whether there is an overfitting problem during training, as shown in Figure 19. As shown in Figure 19, both the training loss and the validation loss are decreasing throughout the training process, which indicates that the model is learning and gradually optimizing. The curves of training loss and validation loss are close to each other, and on most epochs, the training loss is slightly lower than the validation loss. In addition, the smoothed training loss and smoothed validation loss curves show similar trends, further indicating that the training process of the model is more stable. The proximity of the smoothed curves suggests that there is little difference in performance on the training and validation sets. Overfitting usually manifests itself as a decreasing training loss, but the validation loss starts to rise after a certain point, indicating that the model performs well on the training data, but generalizes poorly on the validation data. In Figure 19, there is no clear upward trend in the validation loss curve, indicating that the model does not exhibit a significant overfitting problem over this range of training.

#### 4.4.5. Parameters Analysis

We iteratively train the fusion network and the semantic segmentation network and set the number of iterations Y to 5. As the iterative training proceeds, the parameter *β* is gradually increased to better guide the training of the fusion network. We recorded the β values corresponding to the first iteration training to the fifth iteration training to illustrate the superiority of the *β* setting by observing the effect of different *β* values on the quality of the fused images. The effect of different *β* values on the quality of fused images is shown in Table 4.

From Table 4, it can be seen that as the iterative training proceeds, the value of *β* becomes larger and larger, and the value of each evaluation metric increases. This indicates that as the *β* value increases, the fused image retains more semantic information, thus improving the quality of the fused image. It is worth noting that after the fifth iteration of training, the increase in the values of each evaluation index is significantly reduced, which verifies the rationality of choosing five iterations.

### 4.5. Task-Driven Evaluation

The significance of image fusion aims to better serve high-level vision tasks. Therefore, we will perform semantic segmentation on the fused images by comparing them with other methods in order to demonstrate that our fused images not only have superior visual quality, but also contribute to the performance of high-level vision tasks.

To ensure objectivity and fairness, we retrained the segmentation network on the original MSRS dataset for different fusion algorithms [60]. The training and test sets were set up with the exact same configuration used to train our network. First, we generate the corresponding fused images using each of the seven fusion methods. Then, nine segmentation models were trained on the visible, infrared, and seven fused image training sets, respectively, each trained for 90,000 steps. Our method uses joint training of fusion and segmentation networks, and the segmentation network is also trained for 90,000 steps. The segmentation performance is measured by the pixel intersection over union (IoU), which indicates the degree of overlap between the predicted results and the true labeling, with larger values indicating better segmentation performance. Table 5 shows the segmentation results of our method with other methods on each category. As shown in Table 5, our method achieves the highest IoU values in all categories except for the category of car stop, where it lags behind RFN-Nest by a small margin and ranks first in the mean pixel intersection over union (mIoU). 

We attribute the advantage to two aspects. On the one hand, our fusion network fully incorporates complementary information from infrared and visible images. The complementary information helps the segmentation model to fully understand the imaging scene, which is an important reason why fusion can improve the segmentation performance. On the other hand, guided by semantic loss, our fusion network fully preserves the semantic information of the source images. The rich semantic information enables the segmentation network to describe the imaging scene more accurately, which improves the accuracy and stability of segmentation.

Compared to SeAFusion, although our mIOU improvement is small, the enhancement in detail and quality does help to improve segmentation accuracy in the segmentation task. In the subsequent experiments comparing network operation efficiency, our method has the fastest runtime on all three datasets. This advantage is attributed to our optimization in network design, which significantly improves the overall efficiency of the model by combining Swin Transformer’s multi-head self-attention mechanism and depth-wise convolution to effectively capture global and local features of the image and reduce the amount of computation. This combined improvement not only gives our model a significant advantage in processing speed, but also improves the segmentation accuracy, even though the improvement in mIOU is limited, which shows the superiority and potential of our method in practical applications.

**Figure 20:**
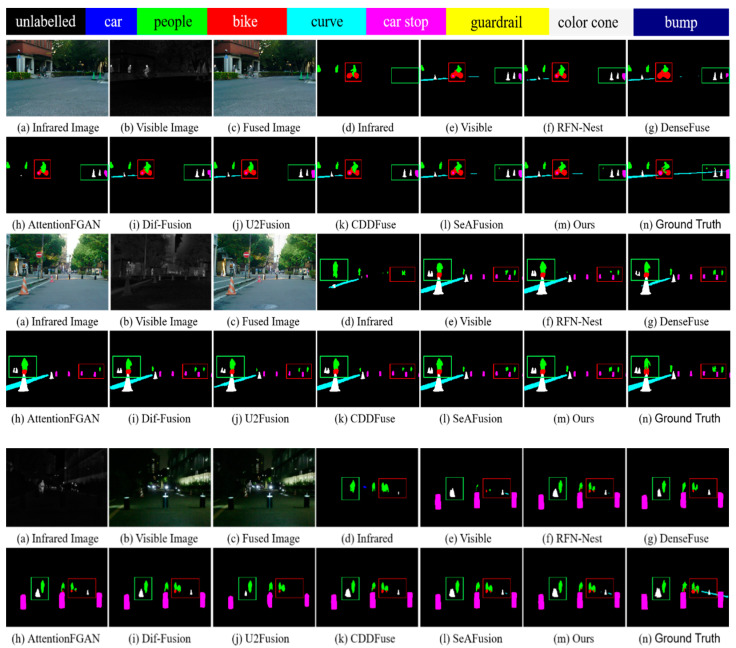
Segmentation results for infrared, visible and fused images in MSRS dataset. The segmentation model is retrained on the infrared image set, visible image set and fusion image set. Each two rows represent one scene, from top to bottom: 00126D, 00366D, and 00752N.

In addition, we provide some visualization examples to show the segmentation results for infrared, visible and different fused images. These segmentation results are obtained with the Deeplabv3+ segmentation model, as shown in Figure 20. Observing the results, it can be noticed that the infrared images provide more information about the main targets such as pedestrians, while the visible images provide a better description of the background, such as cars, bikes and color cones. A good fusion algorithm can fully integrate the complementary information of the source images to achieve a more comprehensive description of the imaged scene. It is worth mentioning that our fusion network fully integrates the complementary information of the source images and effectively integrates the semantic information required for semantic segmentation, and thus our segmentation model can produce more accurate segmentation results. For example, in scene 00126D, we successfully perform a complete segmentation of pedestrians and accurately segment the number of pedestrians. In scene 00366D, we further improved the accuracy of segmenting pedestrians, and were able to accurately capture the pose and contour of pedestrians. And in scene 00752N, we have more comprehensively completed the segmentation of ground curves and accurately extracted the details of the curves.

### 4.6. Ablation Studies

#### 4.6.1. Super-Resolution Network Analysis

**Figure 21:**
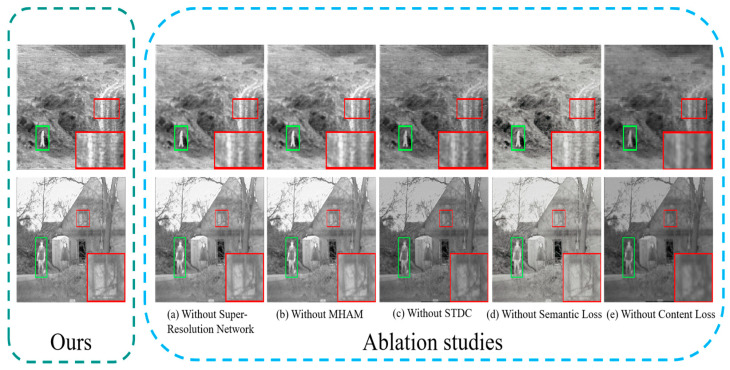
Comparison of visualization results from ablation studies.

Our fused images not only exhibit exceptional visual quality but also contain rich and clear edge and texture information, primarily due to the enhancement of source image quality and details by the super-resolution network. We selected the Duine_sequence_7402 and meting003 images from the TNO dataset for an ablation study, with the visualization results shown in Figure 21. To verify the unique role of the super-resolution network, we directly input the source images into the fusion network for fusion training. It can be observed in Figure 21a that the texture details within the red magnification box appear blurred. This indicates that without the integration of the super-resolution network, the fusion network cannot fully retain the texture information of the source images, leading to a decline in the visual quality of the fused images. We also present the segmentation results of the ablation study in Table 6. We provide the pixel intersection over union (IoU) for cars, people, and bikes, along with the mIoU for all categories. Notably, we observed a significant decrease in the mIoU value without the super-resolution network, suggesting that the super-resolution network plays an important role in facilitating high-level vision tasks.

#### 4.6.2. Multi-Branch Hybrid Attention Module Analysis

The key component in our super-resolution network is the MHAM, which enhances the network’s ability to understand and represent multi-modal features, which in turn produces higher quality super-resolution images. For this reason, we conducted an ablation study of MHAM, and the visualization results are shown in Figure 21b. We can find that with the removal of MHAM, the fused image can maintain the proper intensity distribution, but cannot effectively preserve the texture details in the source image. This is due to the fact that the same super-resolution network may have clarity enhancement for visible images, but no significant improvement for infrared images. In addition, it can be found in Table 6 that the segmentation performance of the fused image without the addition of MHAM shows a decreasing trend. This fully confirms the necessity of introducing MHAM in the super-resolution network.

#### 4.6.3. Comprehensive Information Extraction Module Analysis

A key part of our fusion network is the STDC module, which enhances the network’s ability to characterize fine-grained details. In order to verify the important role of the STDC module, we conducted ablation experiments on the STDC module, and the visualization results are shown in Figure 21c. It can be found that without the addition of the STDC module, the fusion network will not be able to effectively extract the complementary information of the source image. This is manifested in the smoothing of texture details and the weakening of salient targets. In addition, from Table 6, it can be seen that if the fusion network lacks the STDC module, the fused image generated is also somewhat limited in improving the performance of the downstream segmentation task.

#### 4.6.4. Semantic Loss Analysis

We introduce semantic loss to guide the fusion network to fully retain the semantic information in the source image to improve the performance of the fused image in the segmentation task. To further investigate the impact of semantic loss, we perform an ablation analysis and display its visualization results in Figure 21d. Through the ablation study, we found that without the guidance of semantic loss, the fusion network is unable to retain meaningful information of the source image in a targeted manner. This is manifested in the color shift and luminance imbalance of the fused image. In addition, as can be seen from the segmentation results in Table 6, the lack of semantic loss guidance seriously affects the performance of the fused images in the segmentation task.

#### 4.6.5. Content Loss Analysis

The role of content loss is to guide the fusion network to fully integrate the complementary information of the source images so as to generate a fused image that conforms to human visual perception. To further investigate the effect of content loss on the fused image and subsequent advanced visual tasks, we performed an ablation analysis and displayed its visualization results in Figure 21e. It is observed that the visual effect of the fused image is significantly degraded by the lack of content loss guidance, and the image color is dark, and the picture quality is severely blurred. Meanwhile, from the segmentation results in Table 6, it can be seen that the performance of the generated fused image on the semantic segmentation task drops drastically when content loss is missing. This further demonstrates that content loss not only guides the network to generate high-quality fused images, but also effectively improves the performance of subsequent advanced visual tasks.

In summary, our approach purposefully achieves the preservation of intensity distribution and texture details while improving the segmentation performance of fused images, thanks to our unique design including the application of MHAM-based super-resolution network, STDC module, and semantic loss.

## 5. Conclusions

In this paper, we propose a super-resolution-based framework for semantically aware infrared and visible image fusion, aiming at generating high-quality fused images and effectively contributing to the performance of high-level vision tasks. On the one hand, we introduce a super-resolution network to enhance the quality and details of the source images so that the fusion network can integrate the features of the source images more accurately, thus improving the quality and realism of the fused images. On the other hand, a multi-branch hybrid attention module is designed in the super-resolution network, aiming to enhance the network’s ability to characterize multi-modal features in order to obtain super-resolution images with superior visual perception. The multi-branch hybrid attention module selectively focuses on important feature regions in different channels and spatial dimensions, enhancing the processing of critical details.

In addition, a comprehensive information extraction module is designed in the fusion network to efficiently process super-resolution images while achieving more comprehensive extraction of fine-grained complementary features. STDC consists of Swin Transformer mixed depth separable convolution. The inside of the STDC the W-MSA is designed to interact with the Dwconv in a bidirectional form to realize the full extraction of local and global information by the network.

In addition, we introduce content loss and semantic loss to jointly constrain the optimization of the fusion network. Specifically, content loss is used to guide the fusion network to fully integrate the fine-grained complementary information of the source images. Semantic loss is used to guide the training of the fusion network through backpropagation, forcing the fused images to contain more high-level semantic information, thus realizing the outstanding performance of the fused images in high-level vision tasks. 

In future research, we will further explore more task-driven fusion methods, such as evaluating the performance of fused images in a target detection task or achieving joint optimization of target detection and image fusion. In addition, we will explore more image super-resolution methods, e.g., exploiting the complementarity between image fusion and super-resolution tasks to achieve joint optimization of the two. Specifically, good fusion results can improve the effect of super-resolution, while excellent super-resolution results also help to obtain better image fusion results.

## Figures and Tables

**Table 1:** Fusion quality evaluation on 25 image pairs from the RoadScene dataset. RED indicates the best result and BLUE represents the second-best result.

Methods	EN	MI	VIF	SF	SD	Qabf
RFN-Nest	7.2978	2.7287	0.7234	0.0288	9.9871	0.3465
DenseFuse	7.1293	2.9048	0.7343	0.0375	9.9172	0.4763
AttentionFGAN	7.1521	2.7203	0.7041	0.0451	10.0182	0.4821
Dif-Fusion	7.4284	3.3072	0.8053	0.0516	10.1067	0.5181
U2Fusion	7.1681	2.7193	0.7053	0.0498	10.0191	0.4901
CDDFuse	7.4396	3.1493	0.8321	0.0523	10.6732	0.5198
SeAFusion	7.5947	3.2389	0.9182	0.0643	10.6648	0.4922
Ours	7.6326	3.2903	0.9191	0.0648	10.6714	0.5215

**Table 2:** Fusion quality evaluation on 25 image pairs from the TNO dataset. RED indicates the best result and BLUE represents the second-best result.

Methods	EN	MI	VIF	SF	SD	Qabf
RFN-Nest	6.8676	2.0234	0.7678	0.0252	9.2451	0.3392
DenseFuse	6.7388	2.1961	0.7653	0.0375	9.1376	0.4346
AttentionFGAN	6.8382	2.7203	0.7441	0.0463	9.3148	0.4821
Dif-Fusion	7.0865	2.6213	0.9248	0.0541	9.4341	0.4594
U2Fusion	6.8915	1.9876	0.7661	0.0486	9.3268	0.4293
CDDFuse	7.1986	2.1886	0.8713	0.0511	9.4152	0.5068
SeAFusion	7.1241	2.7654	0.9512	0.0522	9.4458	0.4876
Ours	7.1832	2.8235	0.9636	0.0534	9.4762	0.5209

**Table 3:** Means and standard deviations of run times of different methods on MFNet, RoadScene and TNO datasets. RED indicates the best result and BLUE represents the second-best result.

Methods	MSRS	RoadScene	TNO
RFN-Nest	0.1924 ± 0.0901	0.1146 ± 0.0224	0.1951 ± 0.0979
DenseFuse	0.2828 ± 0.1532	0.6064 ± 0.0804	0.6791 ± 0.2955
U2Fusion	0.1351 ± 0.1350	0.7484 ± 0.0929	0.5507 ± 0.5186
SeAFusion	0.0115 ± 0.1081	0.0060 ± 0.0025	0.0049 ± 0.0017
Ours	0.0107 ± 0.1069	0.0053 ± 0.0018	0.0043 ± 0.0012

**Table 4:** We perform iterative training on the MSRS dataset. β=γ×(y−1), where γ is set to 1, and y denotes the yth iteration. RED indicates the best result and BLUE represents the second-best result.

	EN	MI	VIF	SF	SD	Qabf
*y* = 1, *β* = 0	5.5134	2.9752	0.8653	0.0337	6.9865	0.5793
*y* = 2, *β* = 1	5.9431	3.3268	0.9173	0.0376	7.4578	0.6263
*y* = 3, *β* = *2*	6.4576	3.7454	0.9662	0.0402	7.9376	0.6763
*y* = 4, *β* = 3	6.7868	4.1128	1.0216	0.0442	8.4275	0.7174
*y* = 5, *β* = 4	6.8096	4.1312	1.0352	0.0451	8.4364	0.7246

**Table 5:** Segmentation performance (mIoU) of visible, infrared and fused images on the MSRS dataset. RED indicates the best result and BLUE represents the second-best result.

	Backgroud	Car	Person	Bike	Curve	Car Stop	Guardrail	Color Tone	Bump	mIoU
Visible	98.27	89.05	59.96	70.05	60.72	71.46	77.91	63.43	75.34	74.02
Infrared	98.23	87.34	70.51	69.27	58.77	68.91	65.57	56.95	72.76	72.03
RFN-Nest	98.51	89.94	72.12	71.41	62.07	74.91	74.86	63.42	79.55	76.31
DenseFuse	98.51	89.35	72.79	71.71	63.41	72.17	74.45	64.89	80.12	76.38
AttentionFGAN	98.50	89.29	72.08	70.99	62.82	73.63	76.12	63.17	77.05	75.96
Dif-Fusion	98.53	90.11	74.22	72.04	64.18	73.72	82.14	63.42	80.95	77.70
U2Fusion	98.52	89.80	72.91	71.12	62.19	72.16	79.25	63.61	77.14	76.38
CDDFuse	98.54	90.15	74.18	72.06	64.14	74.01	82.84	66.38	81.14	78.16
SeAFusion	98.60	90.41	74.30	72.16	65.01	74.08	85.21	66.50	81.40	78.63
Ours	98.63	90.61	74.56	72.45	65.38	74.48	85.38	66.63	81.58	78.86

**Table 6:** The segmentation performance of ablation studies on MSRS dataset. RED indicates the best result and BLUE represents the second-best result.

	Car	Person	Bike	mIoU
Without Super-Resolution Network	90.46	74.38	72.26	78.67
Without MHAM	90.56	74.48	72.37	78.81
Without STDC	90.53	74.45	72.35	78.78
Without Semantic Loss	89.52	71.57	71.05	76.41
Without Content Loss	89.94	73.02	71.65	77.11
Ours	90.61	74.56	72.45	78.86

## Data Availability

The publicly available datasets used in this article can be found at https://github.com/Linfeng-Tang/MSRS (accessed on 1 June 2024), https://github.com/hanna-xu/RoadScene (accessed on 1 June 2024), TNO Image Fusion Dataset (figshare.com).
